# Use of Intravitreal Triamcinolone in the Treatment of Macular Edema Related to Retinal Vein Occlusion

**DOI:** 10.2174/1874364100802010068

**Published:** 2008-04-07

**Authors:** Ashish Sharma, Baruch D. Kuppermann, M. Cristina Kenney

**Affiliations:** Department of Ophthalmology, School of Medicine, University of California, Irvine, USA

**Keywords:** Intravitreal triamcinolone, macular edema, retinal vein occlusion.

## Abstract

**Objective:**

To analyze the increasing trend of intravitreal triamcinolone (IVTA) use in the treatment of retinal vein occlusion-related macular edema.

**Methods:**

We performed MEDLINE/PUBMED searches (September 1984 - December 2007) to identify articles containing the keywords macular edema and triamcinolone. Case reports, reviews and abstracts were identified from references in the reviewed literature. This review focuses on literature published during the past 7 years with more than two-thirds of the articles that we reviewed being printed during the past 5 years. These reports analyzed the success of IVTA in the treatment of macular edema over a 12 month course of time.

**Results:**

The majority of studies suggested promising results for short time periods (4-6 months) after IVTA treatments. However, long term results were not encouraging.

**Conclusions:**

The success of IVTA therapy for short durations has been the impetus for development of sustained release devices to be used in the treatment of macular edema associated with various retinal diseases including edema related to retinal vein occlusion.

## INTRODUCTION

Retinal vein occlusion (RVO) is second only to diabetic retinopathy as the most common form of retinal vascular disease in the United States [1]. The cumulative 10-year incidence of retinal vein occlusion was estimated to be 1.6% in the Blue Mountains population study of individuals aged 49 years and older [2].

Although the pathophysiology of RVO is poorly understood, an important final common pathway is retinal ischemia. Some of the major pathological effects of ischemia on tissue includes breakdown of inner blood–retinal barrier [3], increased VEGF production [4] and generalized inflammation of retinal tissue by release of prostaglandin and interleukins [5].

The clinical course of central retinal vein occlusion (CRVO) and branch retinal vein occlusion (BRVO) may differ but a major cause of visual loss is macular edema in both the diseases [6]. Other causes of visual loss are ischemia and the presence of central hemorrhage.

Therapies in RVO have two aims. One is to reduce macular edema and the other is to prevent neovascularization caused by retinal ischemia. Many therapies such as laser photocoagulation, anticoagulation, hemodilution, laser-induced chorioretinal anastomosis, anti-VEGF treatment, vitrectomy, sheathotomy, and intravitreal steroids have been used to treat these complications [6].

Among these treatments, only anti-VEGF medications, laser photocoagulation, and intravitreal steroids address the pathophysiological mechanisms responsible for vein occlusion. Anti-VEGF medications (Ranibizumab and Bevacizumab) have excellent effects except in cases of severely ischemic retina. Laser photocoagulation improves visual acuity (VA) in patients with BRVO associated macular edema but is ineffective in improving VA in patients with macular edema associated with CRVO [7]. Overall, laser treatment of perfused macular edema only minimally improves vision and offers little hope for patients with poor pretreatment visual acuity. The benefits for ischemic macular edemas are still not known.

Reports have suggested a role for surgical interventions in the treatment of retinal vein occlusion but results of randomized clinical trials are not favorable [8-10].

Being a less traumatic option of treatment, intravitreal steroids are gaining popularity. At this time intravitreal triamcinolone (IVTA) is the most frequently used steroid. Small case series confirmed the visual benefit of IVTA in both perfused and ischemic macular edema. Studies have revealed that it reduces macular edema in both CRVO and BRVO [10, 11]. Greater effects were observed on nonischemic CRVO than ischemic CRVO [12].

There are some complications of IVTA such as increased intraocular pressure (IOP), cataract formation [13], and sterile endophthalmitis [14]. A major limiting factor of intravitreal steroids is their short duration of action. Many studies revealed that effects of IVTA treatment persist for only 4-6 months [15].

## RESULTS

### Best Corrected Visual Acuity

Three of four studies showed improved best corrected visual acuity (BCVA) for approximately 3 months after IVTA injection but there was a subsequent decline in the vision at 1 year. Table **1** shows the BCVA at follow up visits of 1, 3 and 12 months.

### Central Foveal Thickness

Significant improvement in the central foveal thickness was reported in macular edema due both to BRVO and CRVO after IVTA injections [16-18, 20-24]. Table **2** shows that the central foveal thickness as measured by optical coherence tomography (OCT) dramatically decreases through the 3^rd^ month post treatment. However at the 6 month visit there is no significant difference from the baseline thickness and in most of the studies the macular edema had returned.

### Need to Repeat Injection

Most of the studies showed that IVTA injections needed to be repeated between the 4^th^ and 12^th^ months during the follow up period [16-18].

### Complications

In the published literature, the most common complication of IVTA treatment was a transient rise in IOP, and was controlled with topical medication [26-31]. Wingate and coworkers reported that approximately 30% of the study group develop a significant rise (≥5 mm Hg) in IOP above baseline during the first 3 months [30]. Other reported complications were cataract progression and pseudohypopyon [27, 31, 32]. Progression of nuclear sclerotic cataract was reported in 22% of an older group following a single injection [27]. In the nine published reports of IVTA, a total of 224 eyes in 224 patients have been treated without any reported complication of endophthalmitis [26-31, 33, 34].

## DISCUSSION

The natural history outcome of RVO is very poor [35, 36]. Macular edema associated RVO has always been a difficult condition to treat. Until recently there has been no proven treatment of this pathology. The Central Vein Occlusion Study Group showed that there was no significant difference found in VA between laser-treated and untreated eyes at any follow up time points [35].

There are several case reports showing short-term reduction in macular edema due to CRVO and BRVO as measured by OCT following IVTA [11, 23, 37, 38]. Jonas *et al*. reported significant improvement in VA in both eyes after injection with 25 mg of IVTA in a patient with macular edema due to CRVO [39]. They found decreased fluorescein leakage and did not find any significant complications except mild cataract progression and transient increase in IOP. Park *et al. *reported significant anatomical and functional improvement in 10 eyes with macular edema due to nonischemic CRVO. In their study of 4mg IVTA injections and an average follow up of 4.8 months, they found that 60% of the eyes gained ≥2 lines of VA [23].

Greenberg *et al*. [34] studied a patient with bilateral macular edema associated with CRVO. After an intravitreal injection of 4 mg TA, there was significant improvement in the acute CRVO eye but no improvement in VA in the eye with chronic CRVO. A second injection was performed due to decline in the VA after 6 months. OCT showed a decrease of macular edema with restoration of normal macular anatomy in both eyes. Fortunately, there was no elevation of IOP in this patient.

Bashshur [40] studied VA changes in 40 eyes with macular edema due to nonischemic CRVO. Twenty eyes were treated by a single intravitreal injection of 4 mg of TA, and the rest were only observed for the natural course of the disease. Baseline BCVA was from 20/50 to 20/200. Over 10 months, 12 (60%) of the 20 treated eyes had a final visual acuity of 20/40 or better, while only 4 (20%) of the 20 eyes in the observation group had a final visual acuity of 20/40 or better.

Ip & Kumar [37] injected 4 mg IVTA in two patients with macular edema from CRVO and observed an improvement in BCVA in both cases. In one case, concerning a patient with nonischemic CRVO, the improvement lasted until the 6 month follow up visit. In the other case, involving ischemic CRVO, the effect did not last and BCVA after 3 months was worse than before the injection.

Chen and associates [25, 41] reported a favorable response to IVTA in a patient with ischemic macular edema associated with BRVO while the study by Jonas and coworkers revealed no improvement in ischemic macular edema in their 2 patients. In their prospective comparative nonrandomized study [42], Jonas treated 10 eyes with 20 mg to 25 mg IVTA for macular edema associated with BRVO. Their study revealed significant improvement in VA at one month post injection but the long term effect was not reported. Other studies revealed a decline in the BCVA after initial improvement [23, 34, 43, 44].

The most likely cause of the short duration of action of IVTA is elimination of the drug by diffusion. Following intravitreal injection of 4 mg, measurable levels of TA have been detected in aqueous humor up to 3 months [15]. In addition, triamcinolone has been found in aqueous up to 1.5 years following single injections of 20 to 25 mg [45].

## SUMMARY AND FUTURE

IVTA is frequently used for the treatment of various intraocular neovascular and edematous conditions such as RVO and diabetic retinopathy. Typically, steroids are administered intravitreally because steroids given as drops, systemically, or injected into the subconjunctival and sub-Tenon’s space do not reach high enough concentrations to be effective. Additionally, corticosteroids used systemically for prolonged periods of time increased the risk for systemic side effects.

Studies demonstrated the efficacy of IVTA in reducing macular edema in both CRVO [23, 34] and BRVO [11, 38, 41]. In a majority of these reports, there is a short-term effect of improved retinal thickness, decreased exudation and improved visual acuity, but long-term results were not favorable. It is probable that patients with an initial improvement but subsequent decline in BCVA after IVTA may benefit from repeated treatments but re-injections always involve increased risks and frequent follow up. Steroid implants would perhaps obviate this by providing the edematous macula a lower but steady dose of steroid. At the present time there are ongoing clinical trials to assess the efficacy of intravitreal steroid implants.

The only FDA approved steroid implant Retisert (Bausch & Lomb, Rochester, NY) contains fluocinolone acetonide within a tiny drug reservoir (0.59 mg) which delivers sustained levels into the vitreous cavity and is sutured to the sclera through a trans-pars plana incision. In 2005, the FDA approved the Retisert implant for the treatment of chronic noninfectious uveitis affecting the posterior segment of the eye [46]. This implant releases drug at an initial rate of 0.6 μg per day, with dosage decreasing after the first month to a steady rate of 0.3 μg to 0.4 μg per day for approximately 30 months. The most common serious adverse events in the implanted eyes were cataract development requiring extraction and IOP increase. Ninety-five percent of phakic implanted eyes needed cataract surgery, and 35% of implanted eyes experienced increased IOP. A filtering procedure was needed in 28% of implanted eyes, and explantation of the insert was done in 5% of eyes to manage IOP. This fluocinolone acetonide implant also has been studied in a multicenter, randomized, controlled clinical trial for the treatment of diabetic macular edema (DME). Patients were randomized 2:1 to receive either a 0.59 mg fluocinolone acetonide implant or standard of care, which were either repeat laser treatments or observation.

The Medidur implant (Alimera Sciences, Alpharetta GA) also contains fluocinolone acetonide but is much smaller than the Retisert implant. Although the Medidur is a reservoir implant, it is injected into the vitreous cavity with a 25 gauge syringe and is not sutured to the eye wall, therefore, it is allowed to float freely in the vitreous space. Medidur has two models; one is designed to last approximately 3 years and other lasts approximately 18 months. Enrollment in the phase 3 FAME (Fluocinolone Acetonide in Diabetic Macular Edema) trial, which is evaluating a daily dose of 0.2 μg and 0.5 μg of fluocinolone acetonide to the retina, has been completed. FAME is a double-masked, randomized, multicenter study involving more than 900 patients in the United States, Canada, Europe and India. Safety and efficacy will be assessed at 2 years, and patients will be followed for 3 years. Results from this trial will help to determine if Medidur FA is effective and capable of reducing the steroid induced side effects seen in the Retisert trial.

There is also recent interest in the Posurdex drug delivery system (Allergan, Inc., Irvine, CA) which is a sustained delivery formulation of dexamethasone. The Posurdex implant is biodegradable, unlike the Retisert and Medidur implants, and undergoes hydrolysis with degradation to lactic acid and glycolic acid, two naturally occurring metabolic by-products that are then further broken down to water and carbon dioxide. The implant is inserted into the vitreous cavity and floats freely in the vitreous base. Two different dexamethasone dose implants were evaluated in a 6 month, Phase 2, multicenter, randomized clinical trial [47].

The 315 patients in the Posurdex trial had persistent macular edema due to either diabetic retinopathy (n=172), RVO (n=102), Irvine-Gass syndrome (n=27) or uveitis (n=14). In each patient, 1 eye was randomized to treatment with either a 350 μg dose of Posurdex, or a 700 μg dose of Posurdex. At the primary endpoint (day 90 of the study), 2% of the patients who were implanted with the 350 μg dose of Posurdex had an increase in IOP of 10 mm Hg or more from baseline, compared with 2% of patients in the 700 μg group and 1% of patients in the observation arm. All were managed with either observation or topical IOP-lowering medication. No patient required any surgical intervention to control IOP. Cataracts were present in 15% of the 350-μg group, 17.8% of the 700 μg group and 12.4% of the observation group (P<0.001 *vs* observation). The efficacy results showed a dose response curve that was observed for all subsets of patients based on the underlying cause of macular edema. Overall, 18.1% of eyes in the 700 μg group showed a three line improvement in BCVA at 180 days compared to 7.6% in the observation group and 14.6% in the 350 μg group. Clinically and statistically significant reductions in macular thickness by OCT and leakage by fluorescein angiogram were also observed in a dose response fashion. Phase 3 trials are underway evaluating the Posurdex implant for patients with either DME or macular edema caused by RVO.

The prospective, randomized, double-masked STRIDE (Sustained Triamcinolone Release for Inhibition of Diabetic Macular Edema) trial assesses the safety and tolerability of the I-vation TA (SurModics, Eden Prairie, MN) in 30 patients. Dugel and colleagues reported results of a 6 month interim analysis. In the study, patients were randomized to either a slow-release or fast-release implant containing 925 μg of TA. At 6 months, the proportion of patients with BCVA of at least 70 ETDRS letters increased from 14% to 46% in the slow-release group and from 18% to 41% in the fast-release group. 8% of patients in the slow-release group and 18% in the fast-release group gained more than 15 letters. Macular thickness improvement was reported with the use of both implants. Mean IOP increased from 13.9 mm Hg to 16.1 mm Hg in the slow group and from 14.3 mm Hg to16.4 mm Hg in the fast group at 6 months. The patients in the STRIDE study will be followed for 3 years.

The National Eye Institute sponsored study called the Standard Care *vs* COrticosteroid for REtinal Vein Occlusion (SCORE) trial is a multicenter, randomized, Phase III trial to compare the effectiveness and safety of standard care versus IVTA injection(s) for the treatment of macular edema associated with CRVO and BRVO. As of February 29, 2008 when enrollment was concluded, a total 682 subjects with CRVO (271) or BRVO (411) had been randomized in a 1:1:1 ratio to one of three groups: standard care, IVTA 4 mg, or IVTA 1 mg. Follow up examinations are every 4 months for 3 years and will collect ophthalmic data, including VA, IOP, OCT and fundus photography. Fluorescein angiography will be performed at 4, 12 and 24 months. Based on protocol-specific guidelines, repeat intravitreal injections of TA and repeat laser treatment will be provided as clinically indicated. The primary outcome is improvement by 15 or more letters from baseline in best-corrected ETDRS visual acuity score at the 12 month visit. Secondary outcomes include changes from baseline in best-corrected ETDRS visual acuity score, changes in retinal thickness as assessed by stereoscopic color fundus photography and OCT and adverse ocular outcomes.

Results of the SCORE trial will provide answers regarding the use of IVTA in both CRVO and BRVO.

## Figures and Tables

**Table 1:** Summary of Improvement of Best Corrected Visual Acuity After IVTA Injections

Baseline BCVA	1 Month After IVTA	3 Months After IVTA	12 Months After IVTA	Ref.
20/400	20/300	20/300	8/200	[16]
20/100	20/50	20/50	20/70	[17]
20/300	20/166	20/130	20/270	[18]
worse than 20/50	better than 20/50	better than 20/50	better than 20/40	[19]

**Table 2:** Summary of Central Foveal Thickness After IVTA Injections

Baseline Central Foveal Thickness	1 Month After IVTA	3 Months After IVTA	6 Months After IVTA	Ref.
590 µm	212 µm	193 µm	281 µm	[12]
400 µm	228 µm	256 µm	352 µm	[25]
468 µm	310 µm	311 µm	365 µm	[20]
476 µm	329 µm	389 µm	498 µm	[21]

µm = micron.
